# “Cut and Cover”: A Case Series of Dual Modality Treatment With Stricturotomy and Stenting for Inflammatory Bowel Disease-Related Strictures

**DOI:** 10.7759/cureus.91190

**Published:** 2025-08-28

**Authors:** Matthew J Miller, Abhishek Satishchandran, Jeffrey Berinstein, Peter D Higgins, George Philips

**Affiliations:** 1 Gastroenterology and Hepatology Division of the Department of Internal Medicine, University of Michigan, Ann Arbor, USA

**Keywords:** adult ibd, balloon dilation, crohn’s disease (cd), dilation, electrocautery technique, intestinal stricture, lumen-apposing metal stents (lams)

## Abstract

Strictures are common in inflammatory bowel disease (IBD) and are managed medically and endoscopically, if feasible, due to the risk of surgical complications. While endoscopic balloon dilation (EBD) is often successful, the need for repeat dilation and subsequent surgery is common. Endoscopic stricturotomy (ESt) has gained popularity but has been limited by frequent post-procedural bleeding. We hypothesized that lumen-apposing metal stent (LAMS) placement ("cover") after ESt ("cut") could prevent bleeding and re-stenosis, and we tested the feasibility of this "cut and cover" technique. This retrospective study includes five patients (mean age 49 years) at Michigan Medicine who underwent ESt followed by LAMS placement for Crohn's-related strictures over a one-year period. Strictures were short (<2 cm) and included anastomotic strictures. All patients had a stent in place for at least one month (median 54 days), with average endoscopic follow-up completed 170 days post-ESt to assess stricture traversability. Immediate technical success was achieved in all cases, with no procedural complications such as bleeding or perforation, although stent migration was noted in 60% of cases by endoscopic follow-up, and one patient was briefly hospitalized for post-procedure abdominal pain. In all four patients who returned for long-term patency reassessment (mean 5.6 months post-ESt), their strictures remained endoscopically traversable. In our small cohort, we found that ESt followed by LAMS placement was technically feasible and demonstrated potential for high rates of clinical and technical success with few complications. Further multicenter studies are needed to confirm the technique's efficacy and safety.

## Introduction

Strictures are commonly seen in inflammatory bowel diseases (IBD), especially Crohn’s disease, with a 10-15% prevalence at diagnosis, increasing to 50% after ten years [1]. Management of strictures is medical, endoscopic, and surgical. Due to characteristics of the stricture, including length and refractoriness to medical and endoscopic balloon therapy, many patients will require surgical management of strictures [1]. Post-operative complications, however, are frequently seen in IBD regardless of age and have been estimated to occur in up to half of patients in some studies [1-3]. Thus, optimizing endoscopic management of strictures has been a major focus in IBD care.

For decades, endoscopic management of strictures has been accomplished primarily via endoscopic balloon dilation (EBD). EBD has a high technical success rate (>90%) and short-term clinical efficacy (>80%), but nearly half of patients have symptom recurrence with frequent need for repeat dilation and future surgery [1,4]. Complications of EBD include perforation and bleeding in approximately 5% of patients [4]. Endoscopic lumen-apposing metal stent (LAMS) placement has been previously explored for primary management of Crohn’s strictures, but its utility has been dismissed due to lower efficacy and a high rate of complications, especially early distal stent migration (reported >50% in some studies) [5,6]. More recently, endoscopic stricturotomy (ESt) has emerged as an alternative, which utilizes an endoscopic knife to mechanically open the stenotic segment of bowel. ESt is theoretically more durable in alleviating luminal fibrotic narrowing, similar to surgical stricturoplasty. Meta-analysis has shown similar technical success and superior clinical success compared with EBD [7]. While rates of perforation are like EBD, an ongoing challenge has been bleeding, complicating at least 10% of ESt in a meta-analysis [7].

Given the prior reported success in using self-expanding metal stents for treatment of bleeding after use of endoscopic cutting tools [8], we were interested in technical and clinical success as well as complication rates when LAMS are used after ESt. We hypothesize that placement of LAMS may serve to tamponade the site of stricturotomy to prevent bleeding complications. Additionally, we hypothesized that intentional, coordinated mucosal injury with ESt followed by stent dilation allows for re-epithelialization at the stent diameter, which may help prevent re-stenosis during the healing process post-ESt. We aimed to evaluate the feasibility of managing IBD-related strictures with a dual modality of endoscopic stricturotomy and LAMS placement, a so-called “cut and cover” technique.

## Case presentation

Methods

Study Population

This is a retrospective study of adults (≥18 years) seen at a single academic tertiary referral center (Michigan Medicine). We identified all patients with a history of inflammatory bowel disease and endoluminal strictures who underwent endoscopic stricturotomy with subsequent LAMS placement from July 1, 2023, through June 30, 2024.

The Institutional Review Board of the University of Michigan approved this study.

Data Collection

All data were collected retrospectively from a prospectively collected endoscopy database. Baseline characteristics included patient demographics, previous therapy (e.g., endoscopic balloon dilation), and specific IBD treatment at the time of stricturotomy with LAMS placement. Procedure-related data included the type of stricture (anastomotic or non-anastomotic), location of the stricture, stricture length and diameter, number of incisions made, and diameter of LAMS used. All procedure-related adverse events were recorded and evaluated. Follow-up data included duration of follow-up, treatment outcomes (i.e., stricture improvement, resolution, recurrence), and need for additional interventions. We used the CARE checklist when writing our report [9].

Definitions

Immediate technical success was defined as conversion of a non-traversable stricture (by single-channel therapeutic gastroscope) to a traversable stricture after stricturotomy and LAMS placement. Long-term technical success was defined by a traversable stricture ≥3 months after stent removal. Short-term clinical success was defined as improvement or resolution of obstructive symptoms, including postprandial fullness, bloating, and/or hospitalizations at the initial follow-up clinic visit or endoscopy for stent removal. Short-term follow-up was the stent indwell time (e.g., the time between stent deployment and follow-up endoscopy for removal). Long-term follow-up was the time between stent removal and follow-up endoscopy to assess patency (≥3 months after removal).

Results

Patient Characteristics

Table 1 shows baseline and procedure characteristics for the five patients who underwent stricturotomy with LAMS placement during the study period. All patients had Crohn’s disease, with two of five (40%) having a prior diagnosis of ulcerative colitis with subsequent Crohn’s of the pouch. All patients returned for endoscopic removal of LAMS, with all but one (4/5) returning ≥3 months after LAMS removal to assess luminal traversability. The mean age was 49 years (range 33 to 68). Two of five patients (40%) were male. Two of five (40%) had anastomotic strictures after ileocolonic resection, two of five (40%) had strictures within an ileal pouch anastomosis, and one (20%) was surgery naïve with a stricturing at the ileocecal valve. Two of five (40%) had previous EBD to attempt dilation of their strictures. All strictures were less than 2 cm. One of five (20%) patients was actively using tobacco at the time of the intervention.

**Table 1:** Baseline patient and procedural characteristics The dimensions given are inner diameter by length. EBD: endoscopic balloon dilation. IBD: inflammatory bowel disease. ICR: ileocolonic resection. IPAA: ileal pouch anal anastomosis. LAMS: lumen-apposing metal stent. TI: terminal ileum.

Age (yrs)	Sex	Tobacco use	Prior bowel surgeries	Active IBD meds	Location of stricture	Prior endoscopic interventions	Stricture dimensions	Travers-able	Incisions	LAMS size used	Dilation post-LAMS
68	F	No	Colectomy with IPAA	Vedolizumab	Pouch	EBD to max 18 mm	8 x 10 mm	No	2	15 x 10 mm	No
43	M	Yes	ICR	Upadacitinib	End-to-side ileocolonic anastomosis	None	7 x 8 mm	No	2	15 x 10 mm	No
36	F	No	Naïve	Infliximab + azathioprine	Ileocecal valve	None	8 x 10 mm	No	3	15 x 10 mm	Yes
65	F	No	Colectomy with IPAA	Infliximab + azathioprine	2; pouch inlet and 30cm proximal in neo-TI	EBD to max 8 mm	10 x 10 mm proximally, unmeasured distally	Proximal no; distal (pouch) yes	3 (each)	15 x 10 mm (x2)	No
33	M	No	ICR	Risankizumab	Side-to-side ileocolonic anastomosis	None	8 x 10 mm	No	3	20 x 10 mm	Yes

Endoscopic Technique

**Figure 1:**
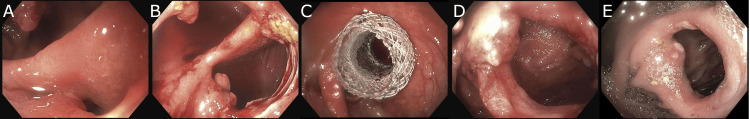
Endoscopic images An example patient is provided, with images A) pre-stricturotomy, B) post-stricturotomy, C) post-stent placement, D) post-stent removal (at first follow-up endoscopy), and at E) patency re-assessment endoscopy (at least three months post-stent removal).

In all cases, a therapeutic gastroscope (GIF-1TH190; Olympus America, Center Valley, PA) was used to perform the stricturotomy and subsequent LAMS stent placement (AXIOS; Boston Scientific Corp., Marlborough, Massachusetts, USA). Fluoroscopy was not used in any case. The gastroscope was advanced, and the stricture(s) were assessed and dimensions measured. If the target stenosis walls were less distinct, the endoscopic ultrasound 20 MHz miniature probe (UM-3R-3; Olympus America, Center Valley, PA) was advanced through the endoscopic channel to estimate the depth of the electrosurgical incision (ESi). In three of five patients, this added modality was utilized to safely optimize the depth of the electrosurgical incision. The ESi was performed at preset sphincterotomy settings (Endocut I mode; effect 2, duration 3, interval 2) with an IT-2 endoscopy knife (Olympus America, Center Valley, PA) in 2 or 3 quadrants at the discretion of the endoscopist based on the endoscopic appearance. After the ESi, the endoscope was advanced across the stenosis to test patency. After stricturotomy, a 15 mm (diameter) by 10 mm (length) or 20 mm by 10 mm stent was advanced freehand through the stricture, and the stent was deployed at the discretion of the endoscopist. Intraprocedural balloon dilation occurred after stent placement in two of five patients at the discretion of the endoscopist. Patients were instructed to follow a modified “stent diet” while the stent was in place (handout on ‘Easy to Chew and Swallow Diet (IDDSI 7EC)’ created by University of Virginia Health System) [10]. All patients had a stent indwell time of at least 36 days (Figure 1, mean 64.6 days [range 36-99]), with removal subject to scheduling and at the discretion of the patient. A follow-up endoscopy was performed at least three months after stent extraction (4/5 patients completed; mean 170 days [range 97-239] post stent removal) and 6-9 months after the index stricturotomy. Example endoscopic images are provided in Figure 1.

Technical Success

Six stents were placed in five patients (Table 1). Stricturotomy was performed in all patients, with most (3/5) patients undergoing ESi in three quadrants. Only one of five patients required the larger diameter 20 mm by 10 mm LAMS placed. Five of five (100%) patients had endoscopies with immediate technical success. There was no evidence of intraprocedural or post-procedural bleeding or perforation in any patient. While stent migration was common by the time of follow-up endoscopy for stent removal (3/5 patients), this was not associated with any complications or need for emergent endoscopy, and all stents were removed at a scheduled follow-up endoscopy. At long-term follow-up endoscopy (completed at a mean of 5.6 months post-stricturotomy [Table 2]), long-term technical success (traversability) was reported in all patients who returned for follow-up.

**Table 2:** Short-term success, long-term success, and complications Short-term follow-up to stent removal was a mean of 64.6 days. Long-term follow-up between stent removal and assessment for luminal patency was a mean of 170.0 days.

Short-term follow-up (days)	Short-term clinical success	Stent migration	Long-term follow-up (days)	Long-term technical success	Complications
99	Yes	Distal	97	Yes	None
36	Yes	None	239	Yes (despite active disease)	Hospitalization-pain
54	Yes	Distal	None	N/A	None
63	Yes	Proximal migrated distally, distal migrated proximally	140	Yes	None
71	Yes	None	204	Yes	None

Clinical Success

**Figure 2:**
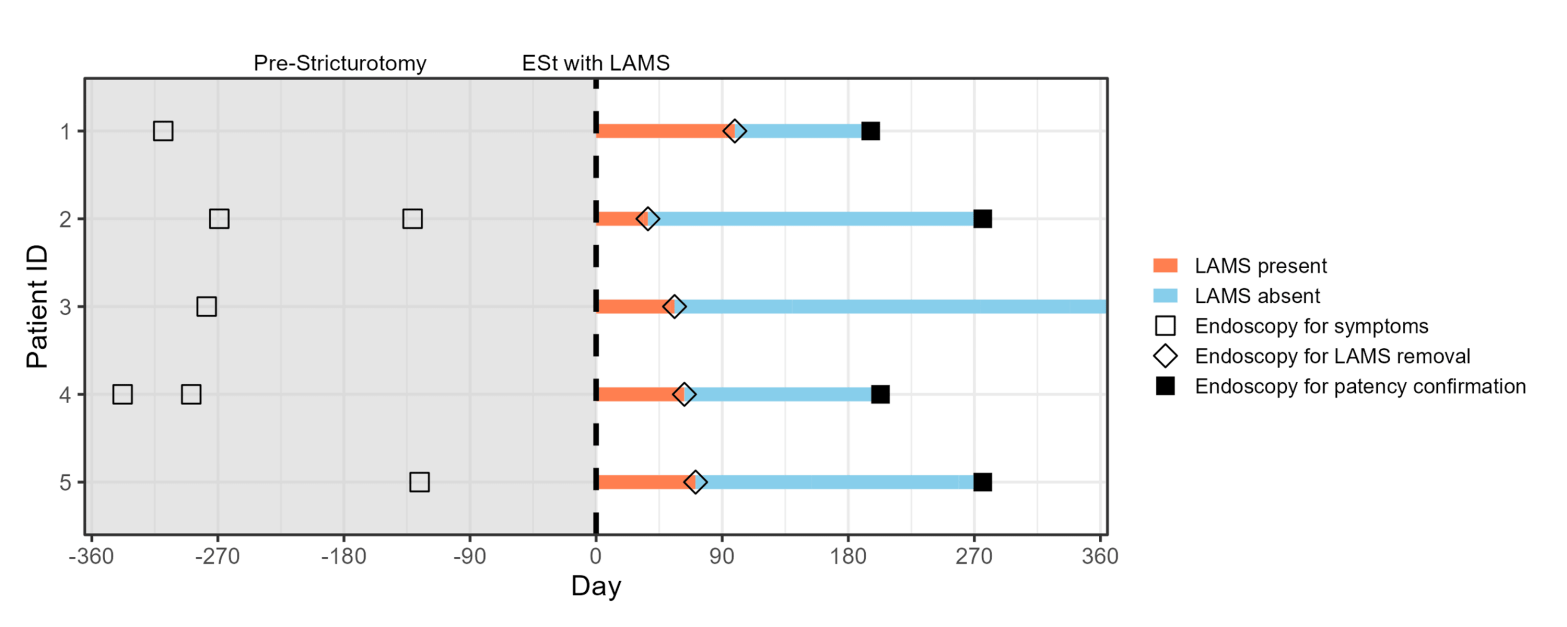
Timeline of endoscopic stricturotomy, LAMS placement, stent removal, and reassessment Endoscopic procedures for symptoms up to one year prior to definitive ESt with LAMS placement (day = 0) were reviewed. No endoscopies were performed for symptoms after ESt with LAMS placement. ESt: endoscopic stricturotomy. LAMS: lumen-apposing metal stent. The image is created by the author.

Immediately post-procedure, one out of five (20%) patients was hospitalized post-procedure for undefined abdominal pain. There was no evidence of perforation, bleeding, stent migration, or other complications with a one-night observation stay and imaging. Otherwise, there were no adverse events that required surgery or endoscopic reintervention. All patients reported symptomatic improvement at short-term follow-up despite frequent stent migration. Among the five patients, they had cumulatively undergone seven endoscopic procedures in the prior year to treat or investigate obstructive symptoms; none required endoscopic treatment for obstructive symptoms in the year following ESt and LAMS placement (swimmer plot, Figure 2).

## Discussion

Among five patients undergoing endoscopic stricturotomy with subsequent LAMS placement, the procedure was technically and clinically successful in all patients. To our knowledge, this is the first study to examine the use of LAMS post-stricturotomy in a series of patients and demonstrate longer-term outcomes. Previous studies have examined the use of LAMS for the treatment of stricturing in Crohn’s disease. In one study [5], stent migration was reported in 80% of patients, with only one of ten patients returning for scheduled endoscopic extraction and others requiring sooner re-intervention or treatment for complications. Despite stent migration in 60% of patients at the time of follow-up endoscopy in our study group, there were no patients who required repeat endoscopy sooner than one month due to stent intolerance, bleeding, or other issues. By performing stricturotomy first, LAMS placement may be safer and more effective in the treatment of Crohn’s strictures.

The patients evaluated in our study were successfully treated with varying states of disease activity and medical therapy. All patients were on advanced IBD therapies at the time of the procedure, with one patient who benefited from a way of reducing obstructive symptoms despite active disease at long-term follow-up endoscopy. Patients with severe ulceration at the site of stricture could not be treated in all quadrants with stricturotomy. Further study is needed to determine if the efficacy of ESt is reduced by a decreased number of incisions. Due to limitations in the ability of LAMS to stretch to accommodate longer strictures, patients with shorter strictures referred to the endoscopist for stricture management would have been preferentially chosen for ESt with LAMS placement and thus would have been included in this study. Strictures >4 cm usually require surgery [1], but the strictures managed in our study were all short (<2 cm); thus, the feasibility of managing longer strictures with this technique requires further evaluation and longer LAMS. Patients in our study experienced no complications aside from one patient briefly hospitalized for post-procedural pain, but this did not require stent removal or re-intervention. The patient who was hospitalized post-procedurally had no evidence of stent migration during hospitalization or at endoscopic follow-up. The cause of the patient's abdominal pain was unclear, but the patient experienced chronic abdominal pain prior to the procedure, and the brief worsening in pain post-procedurally may be related to visceral hypersensitivity. With regard to bleeding, previous studies demonstrated a 10% bleeding rate when stricturotomy was performed alone [7]; however, there were no bleeding-related complications in our small cohort.

Our study provides a novel approach to address refractory strictures in the IBD population by combining stricturotomy with luminal stenting. While stricturotomy has demonstrated greater effectiveness than balloon dilation [7], it has been associated with significant complications. This pilot study demonstrates that endoscopists can utilize a tandem approach of stricturotomy followed by LAMS stenting to address the luminal stricture with a sustained response endoscopically and clinically while averting significant immediate procedural complications. Interestingly, there was a 60% migration rate when the stents were used in tandem. It is unknown as to the optimal duration of stent indwell time since all patients with stent migration had it noted incidentally at the follow-up endoscopy for stent extraction. It is also unknown if stricturotomy increases the risk of stent migration; however, in our series, there was no clear association between the number of incisions and stent migration. Of note, the smaller 15 mm diameter LAMS stent was chosen in most cases, but it is possible that opting for a larger diameter stent, if the proximal small bowel can accommodate it, may reduce the likelihood of stent migration. While our cohort was too small to fully explore this, the one patient with a larger 20 mm stent deployed did not experience stent migration. Overall, our stent migration rate was similar to previous studies but associated with no need for urgent or emergent repeat endoscopy. A second novel concept that was explored in this pilot study was the utilization of the endoscopic miniature ultrasound probe to estimate the safe depth of the ESi. This added modality conceptually provided a more objective assessment and safe stricturotomy.

Limitations of our study include the small sample size and the lack of delineation in the type of stricture (anastomotic vs. non-anastomotic). One clear limitation with this endoscopic technique is the need for a therapeutic gastroscope (Olympus GIF-1T was utilized) for LAMS stent placement. For patients with prior surgical resection, reaching the site of small bowel stricture is not a major concern, but in patients with native anatomy and small bowel stricturing, reaching the site of stricture may not be feasible. However, this is the first pilot study to demonstrate the technical feasibility of this approach and provide prolonged clinical follow-up data.

In summary, in a retrospective study of five patients with IBD strictures treated with a dual modal treatment of ESt with LAMS placement, the procedure did not result in any complications of bleeding or perforation and was technically and clinically successful. The case series demonstrates that this is a feasible endoscopic approach, but more work is needed to define optimal technical parameters, patient selection, and the rate of complications. Stent dwell times such as 6, 8, or 12 weeks could be explored in those who do not experience sooner stent migration. Due to LAMS characteristics, symptomatic, short (<2 cm) strictures should be preferentially chosen. Ideal patient selection would include post-surgical (anastomotic) strictures and fibrotic strictures in quiescent IBD, as these strictures would be unlikely to expand, and endoscopic intervention could spare surgery. Further exploration of this technique would ideally be done in larger, multi-center studies.

## Conclusions

In a small group of patients with IBD-related strictures, ESt followed by LAMS placement was technically feasible and demonstrates potential for high rates of clinical and technical success and few complications. Further multicenter studies are needed to confirm the technique's efficacy and safety.

## References

[1] Rieder F, Zimmermann EM, Remzi FH, Sandborn WJ (2013). Crohn's disease complicated by strictures: a systematic review. Gut.

[2] Fehmel E, Teague WJ, Simpson D (2018). The burden of surgery and postoperative complications in children with inflammatory bowel disease. J Pediatr Surg.

[3] Sacleux SC, Sarter H, Fumery M (2018). Post-operative complications in elderly onset inflammatory bowel disease: a population-based study. Aliment Pharmacol Ther.

[4] Bettenworth D, Bokemeyer A, Kou L (2020). Systematic review with meta-analysis: efficacy of balloon-assisted enteroscopy for dilation of small bowel Crohn's disease strictures. Aliment Pharmacol Ther.

[5] Attar A, Maunoury V, Vahedi K (2012). Safety and efficacy of extractible self-expandable metal stents in the treatment of Crohn's disease intestinal strictures: a prospective pilot study. Inflamm Bowel Dis.

[6] Branche J, Attar A, Vernier-Massouille G (2012). Extractible self-expandable metal stent in the treatment of Crohn's disease anastomotic strictures. Endoscopy.

[7] Jaber F, Numan L, Ayyad M (2024). Efficacy and safety of endoscopic stricturotomy in inflammatory bowel disease-related strictures: a systematic review and meta-analysis. Dig Dis Sci.

[8] Shah JN, Marson F, Binmoeller KF (2010). Temporary self-expandable metal stent placement for treatment of post-sphincterotomy bleeding. Gastrointest Endosc.

[9] Gagnier JJ, Kienle G, Altman DG (2013). The CARE guidelines: consensus-based clinical case reporting guideline development. Glob Adv Health Med.

[10] (2025). University of Virginia Health System: Easy to chew and swallow diet. Virginia Health System.

