# Correction: “Cut and Cover”: A Case Series of Dual Modality Treatment With Stricturotomy and Stenting for Inflammatory Bowel Disease-Related Strictures

**DOI:** 10.7759/cureus.c353

**Published:** 2025-10-09

**Authors:** Matthew J Miller, Abhishek Satishchandran, Jeffrey Berinstein, Peter D Higgins, George Philips

**Affiliations:** 1 Gastroenterology and Hepatology Division of the Department of Internal Medicine, University of Michigan, Ann Arbor, USA

This article has been revised to correct a typo in the title: ‘Stricturetomy’ has been corrected to ‘Stricturotomy’.

